# A Double Whammy Pneumonia: The First Reported Case of Concurrent *Neisseria meningitidis* and SARS-CoV-2 Pneumonia

**DOI:** 10.1177/23247096221111764

**Published:** 2022-07-16

**Authors:** Michael Valdez, Rupam Sharma, Jaspreet Joshi, Harleen Sandhu, Shikha Mishra, Rasha Kuran, Arash Heidari

**Affiliations:** 1 UCLA at Kern Medical Center, Bakersfield, CA, USA

**Keywords:** meningococcal pneumonia, *Neisseria meningitidis*, antibiotics, chemoprophylaxis, pneumonia

## Abstract

Meningococcal pneumonia (MP) is a rare manifestation of meningococcal disease. The MP was first described in 1907 when *Neisseria meningitidis* (NM) isolates were identified in sputum samples obtained from soldiers with pneumonia. Preceding and concurrent viral infections constitute a major risk for MP. During the 1918-1919 influenza pandemic, a significant increase in MP cases were reported in patients with preceding influenza infection. Despite the end of the last H1N1 influenza pandemic in 2010, seasonal influenza infections still pose a risk for simultaneous MP. History appears to be repeating itself with concomitant bacterial and viral coinfection amid the current SARS-CoV-2 pandemic. Herein presented is a unique case of an elderly woman who presented with, to the best of our knowledge, the first reported case of possible concurrent SARS-CoV-2 and MP infections.

## Introduction

*Neisseria meningitidis* (NM) is a Gram-negative aerobic diplococcus that typically colonizes the nasopharynx after inhalation of aerosolized particles containing meningococci. The NM may also be transmitted via direct contact with respiratory secretions. Approximately 10% of the population are asymptomatic carriers.^1,2^ Once local tissues are colonized, NM can invade into the bloodstream, causing numerous forms of meningococcal diseases.^3^ The most common manifestations of NM include meningitis and septicemia. Meningococcal pneumonia (MP) is a rare manifestation of meningococcal disease that was first described in 1907 followed by a significant increase in cases reported during the influenza pandemic in 1918-1919.^4^ The incidence of MP remains unclear but is estimated to be 5% to 15% in patients with invasive meningococcal disease.^5^

Severe acute respiratory syndrome coronavirus 2 (SARS-CoV-2) is a beta-coronavirus that resulted in a worldwide pandemic starting in October 2019.^6^ It leads to a wide range of clinical manifestations, from asymptomatic presentation to a severe form of acute respiratory infection requiring hospitalization and ventilator support. Common symptoms observed between both infections (SARS-CoV-2 and MP) include fevers, chills, myalgias, fatigue, cough, and dyspnea.

Only 344 MP cases have been documented worldwide between 1906 and 2015.^4,5,7^ In the United States, only 3 cases of MP have been reported between 1998 and 2018.^2^ To the best of our knowledge, this is the first reported case of possible concurrent NM and SARS-CoV-2 pneumonia.

## Method

The Institutional Review Board of Kern Medical approved this study. A retrospective review of the patient’s medical records was performed. Literature search was conducted on PubMed and Google Scholar using the following search terms: meningococcal pneumonia, Neisseria meningitidis, antibiotics, chemoprophylaxis, and pneumonia.

## Case Presentation

An 88-year-old Hispanic woman with diabetes mellitus, hypertension, and reactive airway disease presented to the emergency department with a 3-day history of dyspnea. Associated symptoms included productive cough, subjective fevers, and generalized myalgias. The patient had recently returned from a trip to Mexico where she was exposed to multiple family members infected with SARS-CoV-2.

Vital signs at presentation included temperature 36.9°C, blood pressure 84/50 mmHg, heart rate 109 beats per minute, respiratory rate 25 breaths per minute, and oxygen saturation 97% on ambient air. Upon ambulation, patient experienced hypoxia with oxygen saturation 80% on ambient air. Physical examination was pertinent for positive bronchial breath sounds on the right chest.

**Image 1.**
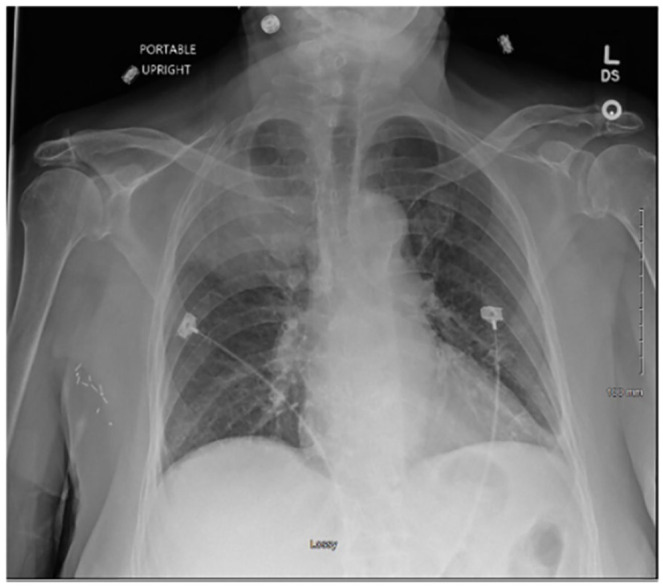
Chest X-ray at initial presentation demonstrating a 7-cm right upper lobe opacity.

**Image 2.**
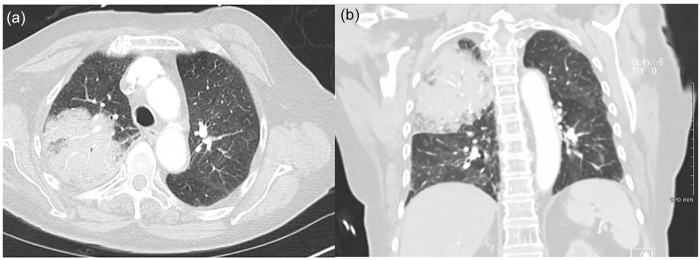
Computed tomography scan at initial presentation, including axial (a) and coronal (b) views, demonstrating large right upper lobe consolidation with air bronchograms, scattered ground-glass opacities, and mediastinal lymph nodes.

Laboratory studies were significant for bandemia of 50%, lactic acid 2.5 mmol/L (reference range 0.4-2.0 mmol/L), procalcitonin 3.6 ng/mL (normal high ≤ 0.50 ng/mL; critical high > 1.99), and elevated inflammatory marker C-reactive protein (CRP) 4.99 mg/dL (normal high ≤ 0.30). Nasopharyngeal swab for SARS-CoV-2 RNA was reactive. Swabs from nares for influenza A and B antigens were negative. Chest radiograph revealed a 7-cm right upper lobe (RUL) opacity (Image 1). Subsequent computed tomography (CT) angiogram of the chest revealed a large RUL consolidation with air bronchograms, scattered ground-glass opacities, and mediastinal lymph nodes with no evidence of pulmonary emboli (Image 2(A) and (B)).

**Image 3.**
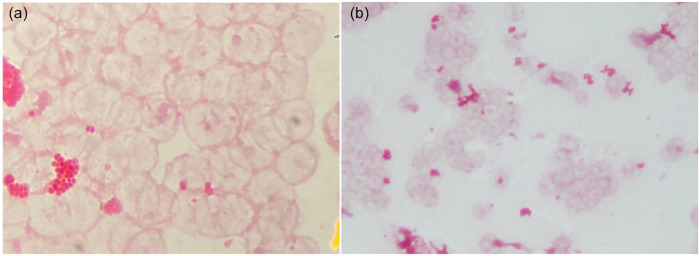
Blood culture (a, b) shown at 60× magnification (a) revealing Gram-negative diplococci.

She was initiated on ceftriaxone 1 g intravenous (IV) every 24 hours and azithromycin 500 mg IV every 24 hours for suspected community-acquired pneumonia. She was started on prednisone 40 mg oral (po) daily in the setting of hypoxia and COVID-19. Bacterial sputum culture was ordered, however was, unfortunately, not collected for reasons that remain unclear. Coccidioidomycosis serology was negative, including nonreactive immunodiffusion for IgM and IgG as well as complement fixation titer <1:2. QuantiFERON tuberculosis gold test was also negative. In addition, 2 sputum acid-fast bacillus smears and cultures were negative. Preliminary routine blood cultures (aerobic and anaerobic bottles) grew Gram-negative diplococci in 1 of 2 bottles from 1 set and final culture revealed NM (Image 3(A) and (B)). Subsequently, ceftriaxone was increased to 2 g IV every 24 hours.

The patient remained alert and oriented and denied any headaches or neck stiffness. Neck was supple with no nuchal rigidity or signs of meningismus. Therefore, suspicion for meningitis was low and lumbar puncture was not performed. Bronchoscopy was considered, however was deferred given the patient’s improvement in symptomatology with antibiotic therapy and in an attempt to reduce potential exposure to coronavirus by avoiding an aerosolizing procedure.

Repeat blood cultures revealed no growth. The patient remained on airborne isolation precautions while in the hospital and infection control was notified of the blood culture growing NM. After 5 days of ceftriaxone therapy, the patient was discharged home with supplemental oxygen at 2 L via nasal cannula for COVID-19-associated hypoxia. Results of susceptibility testing were not available at the time of discharge; however, per the Infectious Disease service recommendations, she was also discharged with 9-day supply of amoxicillin/clavulanic acid 875 mg/125 mg po twice daily to complete a total of 14 days of antibiotic therapy.

**Image 4.**
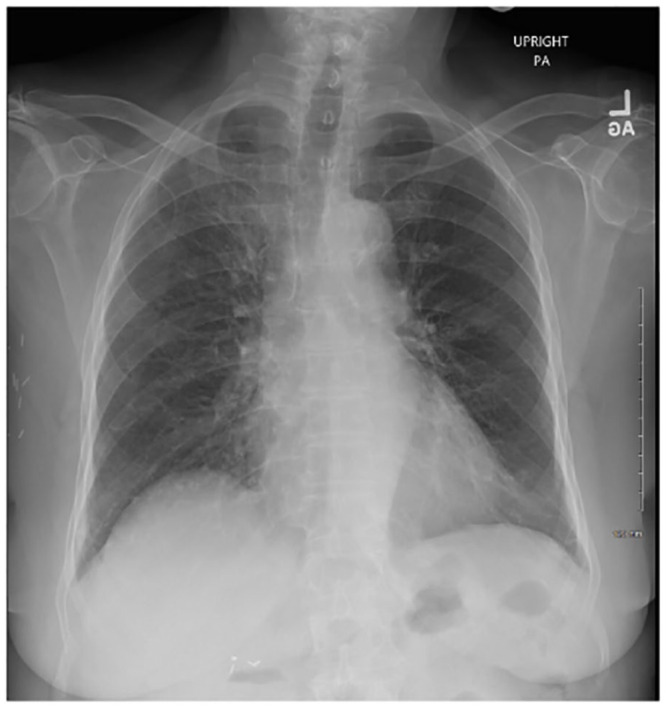
Chest X-ray 3 months after hospital discharge revealing near-complete resolution of previously noted right upper lobe consolidation upon hospital admission.

The NM sensitivities returned approximately 4 weeks after discharge. The isolate was sensitive to ceftriaxone, chloramphenicol, and meropenem but was resistant to ampicillin, levofloxacin, penicillin, and trimethoprim/sulfamethoxazole (Table 1). Patient was seen in the geriatric clinic approximately 3 months after discharge at which time her symptoms had completely resolved and she was no longer hypoxic. Repeat chest X-ray at 3 months demonstrated near-complete resolution of the previously noted RUL consolidation (Image 4).

**Table 1.** *Neisseria Meningitidis* Isolate Sensitivities.

Minimum inhibitory concentration		
Ampicillin	>4	R
Ceftriaxone	≤0.030	S
Chloramphenicol	1	S
Levofloxacin	0.250	R
Meropenem	≤0.060	S
Penicillin	>1	R
Trimethoprim/Sulfamethoxazole	2	R

Abbreviations: R, resistant; S, sensitive.

## Discussion

The clinical presentation of MP is indistinguishable from pneumonia caused by other infectious organisms. Risk factors for MP include older age and preceding viral infection, possibly due to damage to the nasopharyngeal mucosa during a viral illness in an individual already colonized with meningococcus.^1,8,9^ There are 2 proposed pathophysiology pathways for the development of MP, which include the airway pathway and the blood pathway. The airway pathway includes microaspiration of colonized upper airway secretions or inhalation of airborne droplets. The blood pathway includes seeding of the lung after primary bacteremia.

At least 13 serogroups of meningococci have been identified.^5,10^ Serogroups A, B, C, X, Y, and W-135 are associated with disease in humans and serogroups B, Y, and W-135 are most associated with pneumonia.^4,11,12^ Diagnosis is typically made by blood culture positive for NM in the presence of community-acquired pneumonia. The treatment of choice is a third-generation cephalosporin but fluoroquinolones are frequently used as well.^4,7,12^ Mortality is <10% if treated appropriately.

Household contacts and health care personnel exposed to oral secretions or present during intubation require prophylaxis that typically includes rifampin, ciprofloxacin, or ceftriaxone.^8,12,13^ One case series published in 2009 reported 3 cases of ciprofloxacin-resistant NM.^14^ All 3 cases were caused by serogroup B.^14^ The serogroup of this case’s isolate is not known, but given that it was fluoroquinolone-resistant, suspicion for serogroup B is high. It is also noted that our patient’s blood cultures sterilized after 5 days of appropriate antibiotic therapy.

In this particular case, the diagnosis of MP was made based on clinical presentation and radiographic findings consistent with pneumonia and a positive blood culture for NM. One major limitation for this diagnosis is the lack of correlating bacterial sputum culture. In addition, differential diagnosis must also include pneumococcal pneumonia with concurrent meningococcal bacteremia, both of which had favorable response to the ceftriaxone that the patient was receiving. One additional limitation to our hypothesis is whether or not the patient truly had SARS-CoV-2 pneumonia or simply a positive COVID polymerase chain reaction. Although CT angiogram of chest showed minimal ground-glass opacities, the large RUL consolidation is more consistent with bacterial pneumonia and therefore hypoxia may have been due to bacterial pneumonia alone.

The NM as the underlying cause for pneumonia should be considered when blood or sputum cultures identify Gram-negative diplococci. Early recognition is critical to reduce the risk of transmission to close contacts and health care personnel. Given the high mortality rates associated with untreated meningococcal disease, early initiation of appropriate antibiotic therapy is essential in attempting to improve the outcomes of meningococcal disease. Finally, this case may potentially represent history repeating itself in that just as NM pneumonia cases increased during the influenza pandemic in 1918,^4^ to the best of our knowledge this could possibly be the first reported case of concurrent SARS-CoV-2 and NM pneumonia.
